# Recent advances in early stage lung cancer

**Published:** 2021-04-06

**Authors:** Javier Luna, Amalia Sotoca, Pablo Fernández, Celia Miralles, Aurora Rodríguez

**Affiliations:** ^1^Department of Radiation Oncology, Fundación Jiménez Díaz, Madrid, Spain; ^2^Department of Radiation Oncology, Ruber International, Madrid, Spain; ^3^Department of Thoracic Surgery, Fundación Jiménez Díaz, Madrid, Spain; ^4^Department of Medical Oncology, Ruber Clinic, Madrid, Spain

**Keywords:** early stage lung cancer, surgery, adjuvant chemotherapy, stereotactic body radiotherapy, immunotherapy

## Abstract

Treatment of early-stage non-small cell lung cancer has undergone considerable change in recent years. Areas of great interest to researchers include less invasive surgical methods with lower associated morbidity, indications for adjuvant chemotherapy and radiotherapy, the emergence of stereotactic body radiotherapy (SBRT) for peripheral and central or ultracentral tumors, and the probable role of adjuvant immunotherapy following surgery and SBRT, all of which may influence the management of these patients.

**Relevance for Patients:**

At present, the treatment of early stage non-small cell lung cancer is undergoing changes associated with the evolution of existing treatments and the advent of new treatments.

## 1. Introduction

Lung cancer is the leading cause of cancer-related death in the United States among both men and women, with an estimated 154,050 deaths annually [1]. There are over 200,000 new cases of lung cancer each year [2], approximately 85% of which are non-small cell lung cancers (NSCLCs), the predominant histologic subtype. Approximately 16% of NSCLC patients present with early-stage disease (T1-2 N0, according to 8^th^ Edition of the TNM Classification for Lung Cancer) [3], though this rate may increase in the coming years with the introduction of low-dose computed tomographic screening algorithms [4].

Traditionally, Stage-I or II lung cancer has been treated surgically, with a 5-year overall survival (OS) of 72% (stage T1) and 55% (stage T2) [5]. Open surgery (lobectomy or pneumonectomy) has been the surgical approach of choice, though most patients now receive video-assisted thoracic surgery (VATS), which minimizes surgical complications [6].

Patients deemed medically inoperable due to comorbidity or those who refuse surgery have historically been treated with definitive conventionally-fractionated external beam radiotherapy (RT) over 6-7 weeks with generally inferior results to surgery (5-year survival range from 6% to 32%). This is influenced by selection bias, as inoperable patients usually have more medical comorbidities and are older, and the inferiority of clinical versus pathologic tumor staging [7-9].

Since its development in the 1990s at the Karolinska Institute in Stockholm, Sweden, as an adaptation and extrapolation of excellent outcomes from intracranial stereotactic radiosurgery [10], when the control of respiratory motion first became feasible, stereotactic body radiotherapy (SBRT), also known as stereotactic ablative radiotherapy (SABR) has emerged as one of the most significant advances in modern RT. SBRT can deliver high doses of radiation in a few fractions (fx) within the tumor and provide a steep dose drop-off outside the target [11,12]. SBRT has now become the standard of care in early-stage medically inoperable NSCLC, and focus has now turned to maximizing efficacy while minimizing toxicity and to tailoring treatment for high-risk clinical scenarios [13-15].

The role of adjuvant chemotherapy in Stages IA, IB, and II has been increasingly clarified despite remaining doubts as to the management of certain subgroups of patients [16-17]. Prospective studies using molecular markers, targeted therapies, and immunotherapy are currently underway and will likely lead to a deeper understanding of their potential benefit as adjuvant therapies for NSCLC. In addition, the benefit of chemotherapy, targeted therapies, and especially immunotherapy associated with SBRT in subgroups of patients with stage-I or II is also being investigated. Furthermore, other local treatments such as thermal ablation and endoscopic ablative techniques are under clinical evaluation.

## 2. Surgery

Surgery (lobectomy, bilobectomy, and pneumonectomy) remains the standard of care in early-stage (T1-2, N0) NSCLC [18]. To date, these pulmonary resection techniques have been preferred over sublobar resections due to the previous data suggesting inferior survival outcomes with lesser resections [19]. However, sublobar resection (including wedge resection and segmentectomy) can be considered appropriate for patients at high risk for lobectomy who have a small peripheral nodule (ideally 2 cm or smaller). Perioperative morbidity and mortality are reduced and long-term survival is improved when surgical resection is performed by a board-certified thoracic surgeon.

Surgical procedures, both of diagnostic (lung and node biopsies, pleural approach, etc.) and therapeutic intent, have evolved dramatically in recent years as evidenced by the reduced aggressiveness of interventions as well as the degree of lung resection performed, the latter mostly due to the role played by minimally invasive surgery.

VATS was introduced almost three decades ago and has undergone exponential development in the treatment of lung cancer. The standard three-incision approach, including a utility incision of approximately 3 cm to 5 cm [20], has gradually given way to less aggressive techniques. Dr. Diego González Rivas (A Coruña, Spain) and the technique he developed, based on these first thoracoscopic approaches, called “Monoportal VATS,” have been fundamental to the evolution of the technique. Since 2010, when the uniportal approach to major lung resection was introduced, it has become a widespread approach worldwide. The single-port technique provides a direct view of the target tissue. The parallel instrumentation achieved with the single-port approach mimics the maneuvers performed during open surgery. In addition, it is the least invasive approach possible, and by avoiding the use of a trocar, intercostal nerve compression can be minimized. Further development of new technologies such as sealing devices for all vessels and fissures, robotic arms that open within the chest and wireless cameras will contribute to the advancement of the single-port approach as the standard surgical procedure for lung resection in most thoracic surgery departments [21].

As the surgeon’s experience with VATS uniportal lobectomy grows, more complex cases can be performed, thus expanding the indications for single-incision thoracoscopic lobectomy. As a result, VATS has become the most widely used technique in hospitals that treat this type of disease, accounting for almost 80% of the procedures performed.

At present, VATS lobectomy is considered a feasible, safe, cost-effective, and appropriate oncological procedure. In centers, where this technology is available and the surgical team is adequately skilled to perform it safely, VATS should be considered the standard of care for operable lung cancer [22].

Despite its promising future, one of the concerns surrounding VATS is whether we are understaging the patients to offer them minimally invasive techniques such as this one. Palade *et al*. concluded that mediastinal lymph node dissection (MLND) performed with the video-assisted approach is as effective as surgery using the open thoracotomy approach. Furthermore, the video-assisted approach allows better visualization of different lymph node zones [23].

Therefore, both in minimally invasive and open surgery, the role of lymphadenectomy (MLND) provides patients with the most accurate staging and the opportunity for adjuvant therapy if occult metastatic disease is present. The results of the ACOSOG trial indicate that all patients with resectable NSCLC who undergo MLND show no increase in mortality or morbidity [24].

Another technique related to minimally invasive procedures is the technique called “awake surgery.” Recent advances in the treatment of early-stage NSCLC have focused on less invasive anesthetic and surgical techniques. VATS without tracheal intubation is an evolving technique that aims to provide a safe alternative with fewer short-term complications and faster post-operative recovery [25].

Another substantial improvement in surgery came with the advent of robotic VATS surgery, whose best known and most used device, the Da Vinci system, has been applied in different specialties of surgery. Robotic techniques began to be used in gynecological and urological surgery, and later moved on to other types of surgery. Robotic surgery has such advantages as magnified 3D HD vision, improved ergonomics, precision of movement, filtering of physiological tremor, and greater maneuverability (i.e., “7 degrees of freedom”), although initially the procedure also has its drawbacks, such as the inability to palpate lesions, the need for more ports of entry, a longer learning curve as well as higher costs than conventional thoracoscopic surgery. The technique is currently used in procedures ranging from lung biopsies to major lung resection surgery (lobectomies).

Robotic lobectomy is associated with equivalent recurrence-free survival and long-term OS when compared to VATS and the traditional open thoracotomy approach as described in some articles [26]. It is not recommended to abandon open surgery altogether, as it is required in some cases, due either to complex resections or reconstructions of other thoracic structures required or due to the volume of the tumor.

Another situation that has changed is the degree of resection. To date, lobectomy with regulated mediastinal lymphadenectomy remains the gold standard technique for early-stage lung cancer. Sublobar resections are used in patients with high comorbidity as well those with impaired lung function, in whom a lobar resection is not feasible. There are ongoing trials such as the Japan Clinical Oncology Group trial, where the patients with a total tumor size ≤3 cm and a tumor consolidation rate ≤0.5 have shown an excellent prognosis and may be suitable candidates for sublobar resection. If a similar survival of segmentectomy compared to lobectomy is confirmed, sublobar resection is likely to be included in oncology resection procedures [27].

Another important trial currently under way is CALGB/Alliance 140503, which shows that perioperative mortality and morbidity do not seem to differ between lobar and sublobar resection in physically and functionally fit patients with clinical T1aN0 NSCLC [28].

Depending on their final results, both trials may propose an alternative resection to existing approaches to lung cancer in early stages.

All the techniques discussed above must meet the criteria set by the scientific societies supported by the different trials. These trials will determine where surgery is headed and its future role in treatment of early-stage lung cancer.

## 3. Adjuvant Systemic Treatment

Patients with Stage-I and II NSCLC have a significant risk of recurrence and death even after complete surgical resection (R0). Approximately 40-50% of Stage-IB patients and 55% to 70% of Stage-II patients will have recurrent disease despite potentially curative surgery.

• Stage IA

Patients with Stage-IA disease should not receive adjuvant chemotherapy. In a meta-analysis that included five studies with 4600 patients with completely resected NSCLC treated with cisplatin, a trend toward worse survival was observed among patients with Stage-IA disease who received adjuvant chemotherapy [29].

In a National Cancer Database (NCDB) retrospective study involving over 10,000 patients, adjuvant chemotherapy showed no survival benefit among patients with Stage-IA NSCLC and high-risk disease (lymphovascular involvement, visceral pleural involvement, high-grade tumor, sublobar resection, and tumor size) [30].

• Stage IB

The role of adjuvant chemotherapy for Stage-IB patients with R0 resection remains controversial. In general, adjuvant chemotherapy is proposed for patients whose tumors present one or more high-risk features, such as lymphovascular invasion, poor tumor differentiation (including neuroendocrine masses), wedge resection, visceral pleural involvement, and unknown lymph node involvement (Nx). High fluorodeoxyglucose uptake on PET scan (defined as SUV≥10) has also been proposed as a factor conferring high risk.

However, there is no consensus among expert groups because there is no definitive evidence establishing a real improvement in survival. For example, for patients with stage-IB NSCLC, the American Society of Clinical Oncology guidelines do not support adjuvant chemotherapy for routine use due to the lack of definitive evidence establishing improved survival [16]. In contrast, the NCCN guidelines consider observation or adjuvant chemotherapy as appropriate options for patients with early resected NSCLC, depending on the risk factors for recurrence, especially tumor size [17].

No existing studies have clearly identified which patients with Stage-IB disease should be classified as a high-risk group.

Molecular markers may identify patients who have a higher risk of relapse and who could benefit from adjuvant chemotherapy, although use of these markers should be considered in research.

• Stage II

In patients with completely resected Stage-II disease, the use of adjuvant systemic chemotherapy with a cisplatin-based regimen is widely accepted [16,17].

Several clinical trials using platinum-based combinations have demonstrated a clear benefit in Stage-II and -IIIA NSCLC patients with complete resection [31-37].

In the LACE meta-analysis [29], which combined individual patient data from five clinical trials [31-35], a 4-5% improvement in survival was demonstrated at 5 years using cisplatin-based adjuvant schemes.

In a pooled analysis of 4584 NSCLC patients treated with complete resection surgery (R0) with a median follow-up of 5.2 years, adjuvant chemotherapy was associated with a 5.4% decrease in the risk of death at 5 years compared to non-chemotherapy (hazard ratio [HR] 0.89, 95% CI 0.82-0.96) [29]. A statistically significant survival benefit was only observed in patients with Stage-II (HR for death 0.83, 95% CI 0.73-0.95) and IIIA disease (HR for death 0.83, 95% CI 0.72-0.94).

### 3.1. Adjuvant treatment schedules and duration

The standard chemotherapy regimen for early-stage NSCLC patients with R0 resection is a combination of cisplatin with another drug, usually vinorelbine but also gemcitabine, docetaxel, etoposide, or pemetrexed [38-40].

The cisplatin-pemetrexed scheme is used for non-squamous carcinomas, especially adenocarcinomas [40,41].

For the remaining histological subtypes, combinations of cisplatin with vinorelbine, gemcitabine, docetaxel, or etoposide are used [18,39-43].

The addition of bevacizumab to chemotherapy, which is useful in advanced stages, has not demonstrated benefit in early stages [44].

Replacement of cisplatin with carboplatin is not supported by clinical trial data. In general, carboplatin is used when cisplatin is contraindicated and in elderly patients [36].

Cisplatin and carboplatin are usually administered over 4-6 cycles of treatment.

Although in most studies the interval between surgery and the start of chemotherapy was restricted to 6 weeks, an analysis of the NCDB showed a comparable result in patients treated following a longer interval after resection [39].

At the moment, we do not have predictive biomarkers that can be used to aid in selecting the type of chemotherapy to be used and which can be useful to determine which patients with early-stage NSCLC treated with optimal surgery could benefit from adjuvant chemotherapy.

Predictive molecular markers have not been evaluated in prospective studies. For cases with mutation in the epidermal growth factor receptor, there is limited evidence from a meta-analysis [45]. Clinical trials have been conducted but have found no benefit [46]. At present, target agents should not be used in early-stage adjuvant therapy.

Immunotherapy has revolutionized the treatment of several types of cancer, including lung cancer. The excellent results achieved in certain advanced NSCLC trials have facilitated research in early-stage NSCLC. In this setting, promising results were obtained in the pilot study by Forde *et al.*, in which two pre-operative doses of the PD-1 inhibitor nivolumab was administered in adults with untreated, surgically resectable lung cancer. Twenty-one patients were included, of whom 14 (67%) had a pre-operative stage of I-II. Neoadjuvant nivolumab was associated with a major pathological response in 45% of resected tumors. The tumor mutational burden predicted the pathological response to PD-1 blockade [47].

Among the different research groups worldwide, one of the most active in this area is the GECP (Spanish Lung Cancer Group), with several ongoing studies with good preliminary results. Two of these studies stand out particularly:

#### 3.1.1. LINC study

This was a prospective, randomized, and double-blind clinical trial of adjuvant chemotherapy with MEDI4736 (durvalumab) versus placebo in patients with NSCLC with complete resection.

#### 3.1.2. NADIM study

This was a randomized Phase-II study of neoadjuvant chemo/immunotherapy plus adjuvant immunotherapy versus neoadjuvant chemotherapy alone, in potentially resectable NSCLC patients.

### 3.2. Adjuvant RT

In 1998, the systematic review and meta-analysis drawing from nine randomized controlled trials published in the Lancet found that post-operative radiotherapy (PORT) using techniques prior to 1980 had a significant adverse effect on survival in NSCLC, with a HR of 1.21 (95% CI 1.08-1.34) [48] and a 7% decrease in absolute survival concentrated in pN0-pN1 patients. In pN2, a statistically insignificant trend toward better survival with PORT was observed (HR=0.96).

A single-center Phase-III trial not included in the first PORT meta-analysis was designed to evaluate the benefits and drawbacks of PORT in completely resected Stage-I NSCLC. A total of 104 patients who had previously undergone complete surgery for Stage-I lung cancer were randomized to adjuvant RT versus no further treatment. Regarding local control (LC), one patient in the first group had a local recurrence (LR) (2.2%), while in the second group 12 LRs were observed (23%). Overall 5-year survival (Kaplan-Meier) showed a positive trend in the RT group: 67% versus 58% (*P*=0.048) [49].

The Anita Phase-III trial, designed to determine the benefit of adjuvant chemotherapy (cisplatin and vinorelbine), comparing this treatment with observation after surgery in Stages Ib to III, evaluated 840 patients. Each center decided whether to use PORT before initiation of the study. Finally, 232 of 840 patients received PORT (33.3% in the observation arm and 21.6% in the chemotherapy arm). This retrospective evaluation suggested a positive effect of PORT only in pN2 disease [50].

For patients with positive margins after surgery, both with microscopic (R1) and macroscopic (R2) residual disease, the use of RT is beneficial to improve LC. In the event that chemotherapy is also indicated, there is no clear association between treatment sequencing and survival, so in patients with NO R1/R2 resections, concomitant radiochemotherapy treatment may be considered [51].

At present, due to the absence of solid scientific evidence, adjuvant RT should not follow complete resection in patients with early-stage lung cancer, reserving adjuvant radiation therapy for incomplete resections only.

### 3.3. SBRT

Surgery (lobectomy/pneumonectomy with lymphadenectomy) remains the standard treatment for early-stage NSCLC (T1-T2 N0); unfortunately, however, surgery cannot always be performed. Due to the underlying disease of many of these patients, the population aging, increased use of population screening has revealed a substantial percentage of patients who are inoperable or at high surgical risk at the time of diagnosis.

The classic treatment for these inoperable patients has been external RT with conventional fractionation (total dose between 60 and 66 Gy, 1.8 and 2 Gy/day), though outcomes are generally unsatisfactory in terms of LC and survival. In an effort to improve these results, multiple dose-escalation studies were performed, producing contradictory results. In a meta-analysis published in 2016 by Ramroth *et al*. [52], which included 25 randomized studies with 3795 patients, comparing different radical management schemes in NSCLC with or without chemotherapy, the authors concluded that the higher the dose administered (without chemotherapy), the greater the OS.

Looking for a new treatment alternative to achieve a dose escalation, and based on the good results obtained with brain radiosurgery, at the end of the 1990s, Swiss and Japanese doctors presented the first studies of extracranial SBRT. SBRT is a non-invasive, high-precision irradiation technique that allows delivery of ablative doses to lesions located outside the skull with a reduced number of sessions (usually <8).Given the high dose per fraction (much higher than with conventionally-fractionated RT), the biological efficacy of SBRT treatment is also considerably higher, a concept expressed fundamentally in the so-called biological equivalent dose (BED), which is higher than the prescribed absolute dose. In the initial phase of lung cancer, a BED >100 Gy has shown greater benefit in LC of tumors and OS compared to the lower doses [53]. Given the narrow margins required and the high dose gradient, the dose received in the organs at risk (OAR) is lower than with conventional RT, with the resulting reduction in toxicity.

Recently, the results of the Australian Phase III study comparing SBRT to classical RT have been published, including a total of 101 patients with stage-I NSCLC (T1-T2a N0 M0) with histological confirmation, considered inoperable or who refused surgery [54]. Subjects were randomized 2:1 to SBRT (54 Gy in 3 fx or 48 Gy in 12 fx depending on the proximity to the chest wall) or classical RT (66 Gy at 2 Gy/fx or 50 Gy at 2.5 Gy/fx). With a minimum follow-up of 2 years, a lower rate of LR (HR: 0.29, 95% CI, *P*=0.002) and a higher OS (HR: 0.51, 95% CI, *P*=0.02) were observed in the SBRT group.

### 3.4. SBRT for peripheral tumors

SBRT was initially developed for peripheral tumors (PTs). In 2003, Timmerman *et al*. published the results of the first North American Phase-I dose-escalation study in patients with Stage-I NSCLC, in which 37 patients with T1-T2N0 tumors were included in the study, with good LC results and acceptable tolerance with an absolute total dose of up to 60 Gy in 3 fx [55].

Tolerance to treatment is usually good, and asthenia is the most frequent symptom. The development of side effects is related to the proximity of the OAR, and the rate of side effects is higher in CTs.

The efficacy of SBRT in inoperable patients has been demonstrated in multiple prospective Phase-II studies. SBRT is currently the standard treatment for inoperable patients, with excellent LC rates (>85%), cancer-specific survival, and OS at 2-3 years of 70-80% and 50-60%, respectively [56-60].

Multiple fx have been used, making it difficult to compare the results of different studies. Onishi *et al.*, in 2007 [53], published a retrospective study with 257 patients, demonstrating the importance of administering a BED ≥100 Gy to obtain higher LC (91.9%) and OS in this type of patients.

Therefore, at the present time, SBRT is the treatment of choice in patients with early-stage NSCLC that is inoperable or among patients who refuse surgery.

Several attempts have been made to establish the role of SBRT in potentially operable but high surgical risk patients. There are multiple retrospective and observational studies showing that SBRT can be a reasonable alternative option to surgery, especially when compared to sublobar resections, with excellent results in terms of LC, OS, and toxicity.

In 2018, Chen *et al*. published a meta-analysis with 16 studies [61], comparing SBRT and surgery. The OS was superior in the surgery group, and no differences were found in cancer-specific survival, probably due to the difference in patients election (in the SBRT group the patients were older, with worse general condition, and a higher number of comorbidities).

Three Phase-III prospective studies that compared the results of surgery versus SBRT in operable patients (STARS, ROSEL, and ACOSOG Z4099) had to be discontinued due to low recruitment. In 2015, Chang *et al*. [62] published the joint results of 2 of these trials (ROSEL and STARS), with 58 patients included out of the 2000 planned. The estimated OS at 3 years was 95% in the SBRT group versus 79% in the surgery group, with no difference in disease free survival (DFS), LR, regional recurrence, or metastasis. The authors concluded that SBRT could be considered a treatment option in operable patients and called for randomized studies to be conducted.

There are currently three Phase-III studies underway: STABLE-MATES [63], Valor [64], and SABRTooth [65]; in all, SBRT is compared to surgery in high-risk patients. Of these, only the STABLE-MATES trial compares SBRT and sublobar resection.

### 3.5. SBRT for central and ultracentral tumors (UCT)

When we assert that SBRT in early-stage NSCLC produces a high LC with low toxicity and is an excellent alternative in inoperable patients, we refer to PTs, as has been shown in the RTOG 0236 trial [59], in which 54 Gy/3 fx were used. However, this study excluded central tumor (CT) and UCT, in which SBRT has been controversial due to the possibility of increased toxicity.

### 3.6. SBRT for CT

PT differs from central masses in its relationship with OAR. PTs are surrounded by the lung (parallel tissue), while CTs are surrounded by serial tissues (bronchi, great vessels, esophagus, and brachial plexus) with a higher risk of severe toxicity.

The CT is located <2 cm from the proximal bronchial tree (PBT) and immediately adjacent to the mediastinal or pericardial pleura [66]. UCT would also be at <2 cm but contacting OAR.

So far, there are limitations in analyzing the literature on CT: different definitions of CT/UCT, lack of solid data about dose limits for OAR, different SBRT techniques, lack of correlation data between toxicity/dose-volume histogram, and the use of several toxicity scales.

Several studies showed an increase in LC at the expense of increased toxicity in CT.

Timmerman *et al*. [67] used SBRT (60-66 Gy/3fx) in inoperable tumors, achieving a LC of 95% at 2 years and a toxicity ≥G3-5 of 20% and 5 deaths, 4 in CT. Central/hiliar versus peripheral location was the only predictor of toxicity, with an 11-fold increased risk. The percentage of patients without toxicity at 2 years was 83% (PT) versus 54% (CT). Doses ≥20 Gy/fx were not recommended on CT.

Song prescribed 40-60 Gy/3-4 fx on consecutive days in 32 NSCLC patients. Of the nine patients with CT, 33% had G3-5 lung toxicity. No difference in OS regarding the tumor location was reported. The author concluded that a longer fractionation (>4) could be safer [68].

However, optimal fractionation has not yet been established. A 45-Gy/5 fx scheme from 2 MSKCC studies [69,70] showed a lower LC at 2 years, 79% [69] and 63.9% [70], concluding that *BED_10_*<*100 Gy* decreases LC. Chang confirmed this finding and, with a total dose of 50 Gy/4 fx, established the balance between gross tumor volume coverage (*BED_10_≥100 Gy*) and OAR dose (*BED*_3_*≤210 Gy*) as crucial. If dose restrictions cannot be met, the author proposes changing the scheme to 70 Gy/10 fx [71]. None of the schemes in this study was a prognostic factor for OS or DFS.

Haasbeek *et al*. [6], with a risk-adapted dose protocol, 60 Gy/8 fx on 63 CT, showed 93% LC at 3 years, no toxicity ≥G4 and comparable results to PT, showing 60 Gy/8 fx as a safe and effective fractionation.

The ASTRO 2017 guide [15] does not recommend 3 fx but rather 4-5 fx schedules and maximum compliance with both volumetric parameters and maximum OAR dose. If these recommendations cannot be fulfilled, 6-15 fx schedules are recommended.

### 3.7. SBRT for UCT

Toxicity analysis in UCT is controversial, and clinical practice varies widely. While some authors consider the risk of toxicity in this type of tumor to be greater [72-74], others argue that location does not affect toxicity, as evidenced by a high LC and limited toxicity at appropriate fx [75,76].

Corradetti dismisses 50 Gy/5 fx as safe in UCT because of the possibility of fatal hemorrhaging, possibly associated with previous bronchoscopy/biopsy [72]. Stam *et al*. concludes that location matters, with a higher risk of non-cancer mortality observed in tumors within 1 cm from PBT treated with risk-adapted SBRT; non-cancer death in tumors between 1 cm and 2 cm from PBT was not different from non-cancer death in the PT group [73].

However, Chaudhuri *et al*. stated that UCT could be safely treated with 50 Gy/4-5 fx as no differences in LC and toxicity were observed [75]. The limitation of this study is that it only included seven patients.

In the interesting systematic review published by Senthi *et al*. [76] on the outcomes of SBRT for both central and UCT, 20 publications were included in the study, reporting outcomes for 563 CT and UCT patients. Tumor location (central vs. peripheral) did not impact OS. LC rates were ≥85% when the prescribed biologically equivalent tumor dose was ≥100 Gy. Treatment-related mortality was 2.7% overall. Grade 3-4 toxicities may be more common following SBRT for CT and UCT, but occurred in <9% of patients.

Chang, in a retrospective study of 107 patients with CT and UCT treated with 5-fx SBRT, finds no significant differences in OS, LC, or grade ≥3 toxicity between patients with central and ultracentral lung tumors, but recommends caution, given the low number of patients included, until prospective trials are completed [77]. Different studies with SBRT in UCT are summarized in the systemic review and meta-analysis by Rim *et al*. [78] (Table 1).

**Table 1:** Summarizes the studies with complete results with SBRT in UCT in the systemic review and meta-analysis by Rim *et al*. [78].

Study (Publication year)	Patients UCT/CT/PT	SBRT doses (BED_10_/BED_3_)	Prescription	LC 2 years	OS 2 years	Toxicity≥3	Observations
Chaudhuri *et al*. (2015) [75]	7/27/34	50 Gy/4 fx (112.5/258) 50 Gy/5 fx (100/217)	PTV D95%≥100% prescription doses D_max_≤120% prescription doses	UCT 100% CT 90% PT 83.7% *P*=0.64	UCT 80% CT 63.2% PT 86.6% *P*=0.62	UCT 0% CT 3% PT 11.6% 0 G5	No contact with esophagus 86% Primary T Non-compliance with RTOG restrictions in PBT, no increased toxicity
Tekatli *et al*. (2016) [74]	47/0/0	60 Gy/12 fx (90/160)	PTV D95%≥100% prescription doses PTV D99%≥90% prescription doses D_max_≤140% prescription doses 4 consecutive days/1 week OAR doses priority over PTV coverage	UCT 100% CT and PT: N/A	UCT 28.7% CT and PT: N/A	UCT 38% 21.2% G5	Fatal risk factors: Use of anticoagulants Squamous cancer Endobronchial lesion Dmax≥123% prescribed dose Interstitial disease 9% previous radiotherapy Larger T
Haseltine *et al*. (2016) [70]	18/90/0	45 Gy/5 fx (86/180) 50 Gy/5 fx (100/217)	Prescription dose 100% isodoses Alternate-day treatment	77.4% (no differences between UCT vs CT)	63.9% (no differences between tumors<or>1 cm from PBT	UCT 24.8% CT 7% *P*=0.014 G5: UCT 22% CT 0% *P*<0.001	Recent exposure to bevacizumab as a fatal risk factor 94% Primary T
Chang *et al*. (2018) [77]	46/61/0	Several fractionations 50 Gy/5 fx (100/217)	PTV D99%>95% of prescription doses D_max_<120% prescription doses Alternate-day treatment	UCT 95.7% CT 96.6% *P*=0.92	UCT 50.4% CT 57.7% *P*=0.1 Lesser OS with SBRT doses<50 Gy	UCT 8.7% CT 3.5% *P*=0.23 G5: UCT 2.2% CT 3.5% *P=*0.76	Idiopathic pulmonary fibrosis as a fatal risk factor 48% Primary T

UCT: Ultracentral tumor, CT: Central tumor, PT: Peripheral tumor, LC: Local control, OS: Overall survival, PTV: Planning target volume, Fx: Fraction, N/A: Not applicable, SBRT: Stereotactic body radiotherapy, BED: Biological equivalent dose, OAR: Organs at risk, *P*<0.05: statistically significant, *P*<0.001: statistically highly significant

A question that has yet to be fully resolved is whether in UCT we should prioritize tumor coverage or OAR doses. Murrell *et al*. [79] concludes that 60 Gy/8 fx prioritizing OAR tolerance provides an acceptable balance between LC and toxicity. In large tumors and multiple OAR, a more conservative approach (60 Gy/15 fx) prioritizing tumor coverage is reasonable, providing safe doses for OAR with lower LC. The OAR restrictions for UCT treated with SBRT are summarized in Table 2.

**Table 2:** Summarizes the OAR restrictions in different studies.

	RTOG 0813^66^			EORTC LungTech^81^	MDACC^71^		SUNSET^83^					
			
	50-60 Gy/5 fx			60 Gy/8 fx	70 Gy/10 fx		5-6 fx		8-10 fx		15 fx	
					
	Volume	Max volume (Gy)	Max dosepoint (Gy)	Max dose (Gy)	Volume	Max doses	Max Dose (Gy)	Vol Cc (Max dose)	Max dose (Gy)	Vol cc (Max doses)	Max dose (Gy)	Vol cc (Max dose)
Esophagus	<5 cc	27.5 Gy (5.5/fx)	105% PTVpresc	8×5=40	V40≤1 cm	≤50 Gy	40	5 cc (35)	45	5 cc (40)	50.5	5 cc (48)
Heart/Pericardium	<15 cc	32 Gy (6.4/fx)	105% PTVpresc	UR	V45<1 cm	≤60 Gy	62	10 cc (50)	64	10 cc (60)	66	10 cc (62)
Great vessels	<10 cc	47 Gy (9.4/fx)	105% PTVpresc	UR	V50<1 cm	<75 Gy	62	10 cc (50)	64	10 cc (60)	66	10 cc (62)
Trachea and bronches	<4 cc	18 Gy (3.6/fx)	105% PTVpresc	8×5.5=44	V40≤1 cm V50<1 cm	<60 Gy	62	10 cc (50)	64	10 cc (60)	66	10 cc (62)
Spinal cord	<0.25 cc <0.5 cc	22.5 (4.5/fx) 13.5 (2.7/fx)	30 Gy (6 Gy/fx)	8×4=32	V35≤1 cm	<40 Gy	30 32 (PRV 3 mm)		32 34 (PRV 3 mm)		39.5 42 PRV 3 mm	
Brachial plexus	<3 cc	30 Gy (6 Gy/fx)	32 Gy (6.4 Gy/fx)	8×4.75=38 (<0.5 cc)	V50<0.2 cm	<55Gy	32		39		50	
Skin	<10 cc	30 Gy (6 Gy/fx)	32 Gy (6.4 Gy/fx)		V50≤60 cm V40≤120 cm V30≤250 cm	≤82 Gy						
Whole lung	1500 cc 1000 cc	12.5 (2.5/fx) 13.5 (2.7/fx)		UR	Median dose ≤9 Gy V40≤7%		Media<12 Gy		Media<12 Gy		Media<14 Gy	

UR: Unspecified restrictions; Fx: Fraction, OAR: Organs at risk

In summary, SBRT in UCT could be a curative treatment option and its results should be considered in relation to the alternatives, specifically the 8.3% mortality after pneumonectomy or the high mortality from tumor progression after conventional RT [80].

### 3.8. Ongoing trials in CT and UCT

- The RTOG 0813 trial [66] is designed to establish a toxicity profile and determine an optimal dose in a 5 fx scheme for CT. After a median follow-up of 38 months, the 2-year LC was 89% and OS was 68%. With the two highest dose schedules, 11.5 Gy/fx and 12 Gy/fx, the 2-year LC and OS are comparable to that achieved by PT, with acceptable G3+ toxicity. The final results are pending and will be critical in determining the most appropriate dose and schedule.
- EORTC 22113-08113 LUNGTECH, for evaluation of toxicity and efficacy of 8×7.5 Gy in CT [81].
- HILUS, a Phase-II study of SBRT in CT ≤1 cm from PBT. 56 Gy/8 fx [82].
- SUNSET: Phase-I dose-escalation study to define optimal dose and fractionation in UCT [83].

### 3.9. SBRT, chemotherapy, and immunotherapy association

Due to the benefit of adjuvant chemotherapy after surgery in certain types of early-stages NSCLC, this possibility is beginning to be studied in SBRT. In 2019, Foster *et al*. [84] published the results of 24 011 NSCLC (T1-T3N0M0) patients obtained from the NCDB and treated with definitive SBRT from 2004 to 2014.

The association between non-randomized prescription of adjuvant chemotherapy and OS was analyzed for all patients, and a propensity-matched analysis was carried out. A subset analysis was performed for patients with tumors ≥4 cm (*n*=2,323).

Lower OS was obtained in the adjuvant chemotherapy group (*n*=322) following definitive SBRT for T1-3N0M0 NSCLC. No survival benefit for patients with tumors ≥4 cm was determined with chemotherapy.

There is also growing evidence of a clinical synergy between radiation and immunotherapy, with several ongoing trials studying the abscopal effect. This combination has been studied more systematically in the metastatic setting. Recent studies from MD Anderson Cancer Center [85] and the University of Chicago [86] combining immunotherapy and SBRT in a metastatic setting have shown promising results. The PACIFIC trial showed that adjuvant immunotherapy after definitive chemoradiation in Stage-III NSCLC improved PFS [87]. This finding suggests that radiation may work synergistically with immunotherapy, as prior trials in the metastatic setting have shown.

Compared to cytotoxic chemotherapy, immunotherapy might be more tolerable in patients with SBRT, many of them older, frail, or with multiple diseases. NRG Oncology is conducting a Phase-III trial of adjuvant durvalumab after SBRT in early-stage NSCLC, which will test this hypothesis.

### 3.10. Other local treatments for ES-NSCLC

With lower level of scientific evidence so far, other techniques are being developed for the treatment of early-stage lung tumors. SBRT is the most established modality, with extensive evidence demonstrating efficacy in treatment of Stage-I NSCLC. However, its use in patients with underlying pulmonary disease (e.g., fibrosis) or previous radiation treatment could be risked by a higher expected toxicity profile [88].

In these cases, image-guided thermal ablation offers an effective and safe treatment for appropriate palliation and, in some cases, cure of primary thoracic malignancies. Modalities including radiofrequency ablation (RFA), microwave ablation (MWA), and cryoablation use computed tomography guidance for delivery of thermal energy to achieve tumor ablation [89].

All these techniques have demonstrated reasonable efficacy in short-term follow-up, but outcomes beyond 2-3 years are poor. In addition, high rates of major complications are reported, particularly the rates of pneumothorax (up to 60%) among patients; intercostal drains are required in up to 38% of patients [90,91].

For PT, numerous endoscopic ablative techniques are under evaluation. With a more favorable safety profile and the ability to provide diagnosis and staging information potentially within a single procedure, there is a strong rationale for the development of bronchoscopic ablative modalities. Safety remains paramount and must be individually demonstrated in clinical studies for each new device. Numerous clinical studies are under way for several flexible ablation devices, suggesting that clinical evidence for safety and feasibility of multiple bronchoscopic ablation modalities will be available in the near future. So far, RFA appears to be the most advanced bronchoscopic modality, as it is the only modality where experience in clinical studies has been reported [92]. Other bronchoscopic techniques such as bronchoscopic MWA, bronchoscopic thermal vapor ablation, cryoablation, or laser interstitial thermal therapy are under investigation and at the moment are less developed clinically than RFA.

Bronchoscopic therapy is likely, at least initially, to be restricted to patients deemed unsuitable or at high risk for existing treatments [92,93].

## 4. Conclusions

Surgery is the gold standard in the treatment of operable NSCLC. Technical advances such as monoportal VATS, awake surgery, and robotic VATS are providing greater surgical precision and lower morbidity and mortality. The benefit of adjuvant chemotherapy remains unclear in Stage IB and is widely accepted in stage II. The role of molecular markers in defining risk groups, as well as the indication of adjuvant targeted therapies and adjuvant immunotherapy is currently being investigated. In the inoperable patient, SBRT has become the treatment of choice, due to its high LC and low toxicity. Ongoing clinical trials will define the role of SBRT in operable or high-risk surgical patients. SBRT in central and UCT is a huge challenge. There is growing scientific evidence on tumor and organ at risk doses to obtain high LC and acceptable toxicity. Issues such as the association between chemotherapy or immunotherapy and SBRT in early-stage NSCLC patients are under investigation. Other local treatments such as image-guided thermal ablation or endoscopic ablative techniques are currently under clinical evaluation.
